# Bioinspired cobalt cubanes with tunable redox potentials for photocatalytic water oxidation and CO_2_ reduction

**DOI:** 10.3762/bjoc.14.208

**Published:** 2018-09-05

**Authors:** Zhishan Luo, Yidong Hou, Jinshui Zhang, Sibo Wang, Xinchen Wang

**Affiliations:** 1 State Key Laboratory of Photocatalysis on Energy and Environment, College of Chemistry, Fuzhou University, Fuzhou 350002, China

**Keywords:** CO_2_ reduction, cobalt cubane, photocatalysis, water oxidation, water splitting

## Abstract

The development of efficient, robust and earth-abundant catalysts for photocatalytic conversions has been the Achilles’ heel of solar energy utilization. Here, we report on a chemical approach based on ligand designed architectures to fabricate unique structural molecular catalysts coupled with appropriate light harvesters (e.g., carbon nitride and Ru(bpy)_3_^2+^) for photoredox reactions. The “Co_4_O_4_” cubane complex Co_4_O_4_(CO_2_Me)_4_(RNC_5_H_4_)_4_ (R = CN, Br, H, Me, OMe), serves as a molecular catalyst for the efficient and stable photocatalytic water oxidation and CO_2_ reduction. A comprehensive structure–function analysis emerged herein, highlights the regulation of electronic characteristics for a molecular catalyst by selective ligand modification. This work demonstrates a modulation method for fabricating effective, stable and earth-abundant molecular catalysts, which might facilitate further innovation in the function-led design and synthesis of cubane clusters for photoredox reactions.

## Introduction

The direct conversion of solar energy into chemical fuels (e.g., H_2_, CO and hydrocarbons) through water splitting and carbon fixation reactions is a sustainable solution to environmental concerns and long-term access to adequate energy supplies [1–7]. To realize these reactions, extensive studies have focused on the design and synthesis of chemically stable light-harvesting antenna materials and efficient cocatalysts, and their assembly in integrated artificial photosynthetic systems [8–13]. However, such target reactions are typical thermodynamically uphill reactions with large overpotentials, leading to low conversion efficiency. Therefore, the search for suitable cocatalysts to reduce the multielectron involved kinetic barriers for water oxidation and CO_2_ reduction is regarded as a critical step toward artificial photosynthesis, which can boost the photoconversion efficiency (PCE) significantly [14–19].

Molecular catalysts with complex and varied structural motifs are a class of promising catalysts for solar energy conversion, because of their well-controlled functions and tunable nature [20–21]. Their topologies and electron structures can be precisely engineered by ligand design, using the full arsenal of organic chemistry [22–23]. These unique structures benefit not only tailoring their redox and kinetic properties for catalysis, but also providing valuable structural information to understand the mechanistic insights of catalytic behavior [24–27]. In addition, the molecular catalysts can either be dissolved in liquids affording a homogeneous catalytic system [28–29], or immobilized on solid surfaces for application in heterogeneous catalysis [30–33], owing to their molecular nature with flexible ligand architectures [34–35]. In this regard, extensive attention has been contributed to the design and synthesis of molecular catalysts [36]. Unfortunately, most of the high-activity molecular catalysts are typically based on noble metals (e.g., Ru, Ir) [37–40], which seriously restricts their practical applications. Therefore, the development of effective, stable and sustainable molecular catalysts based on earth-abundant elements is highly desirable [41–43].

Inspired by the molecular Mn_4_CaO_5_ cubane of oxygen-evolving complex in photosystem II, there is an emerging number of molecular cubanes with metallic and heterobimetallic cores that are designed and synthesized for photosynthesis and electrochemistry. Cobalt-based molecular catalysts [44], in particular the ones containing a cubical Co_4_O_4_ core were studied extensively as energy conversion catalysts, because of their cubical topology that is structurally analogous to the biological Mn_4_CaO_5_ cubane [45–46]. Driess et al. have reported the smallest possible molecular building block “Co_4_O_4_” cluster with a singly deprotonated dipyridyldiol (LH) as a chelating ligand [47]. Generally, Co_4_O_4_-based molecular catalysts can be easily tuned by ligand design, owing to their molecular nature [48–49]. For example, Hill et al. demonstrated that using polytungstate ligands to stabilize “Co_4_O_4_” cubane units can produce a robust homogeneous catalyst for solar water oxidation [50]. After that, Berlinguette et al. reported that replacing the inorganic ligand with an organic ligand, such as the pentadentate Py5 ligand can also well stabilize the “Co_4_O_4_” unit to catalyze water oxidation [51]. This finding is very important, which means there is ample choice of organic ligand architectures to tailor the electronic properties of the “Co_4_O_4_” unit for catalysis. In this regard, Nocera et al. selected an organic ligand bearing an electron-withdrawing group (fluorine) to optimize the “Co_4_O_4_” cubane unit for electrocatalytic water oxidation [52]. As expected, the resultant catalyst exhibited a larger catalytic current and an earlier onset potential with respect to its analogs without a fluorine functional group. Thus, the control of catalytic properties via molecular design by tunable ligand substitution is essential in the development of Co_4_O_4_-based cubane catalysts. However, most of the researches focused on the oxidative properties of the Co_4_O_4_ core [53], and its use for reduction reactions is rarely covered. Theoretically, the redox potential of Co_4_O_4_ cubane clusters should be tuned by virtue of different ligand substitutions, thus it is highly possible to develop a Co_4_O_4_-based catalyst for reduction applications, such as H_2_ evolution and CO_2_ fixation.

**Figure 1:**
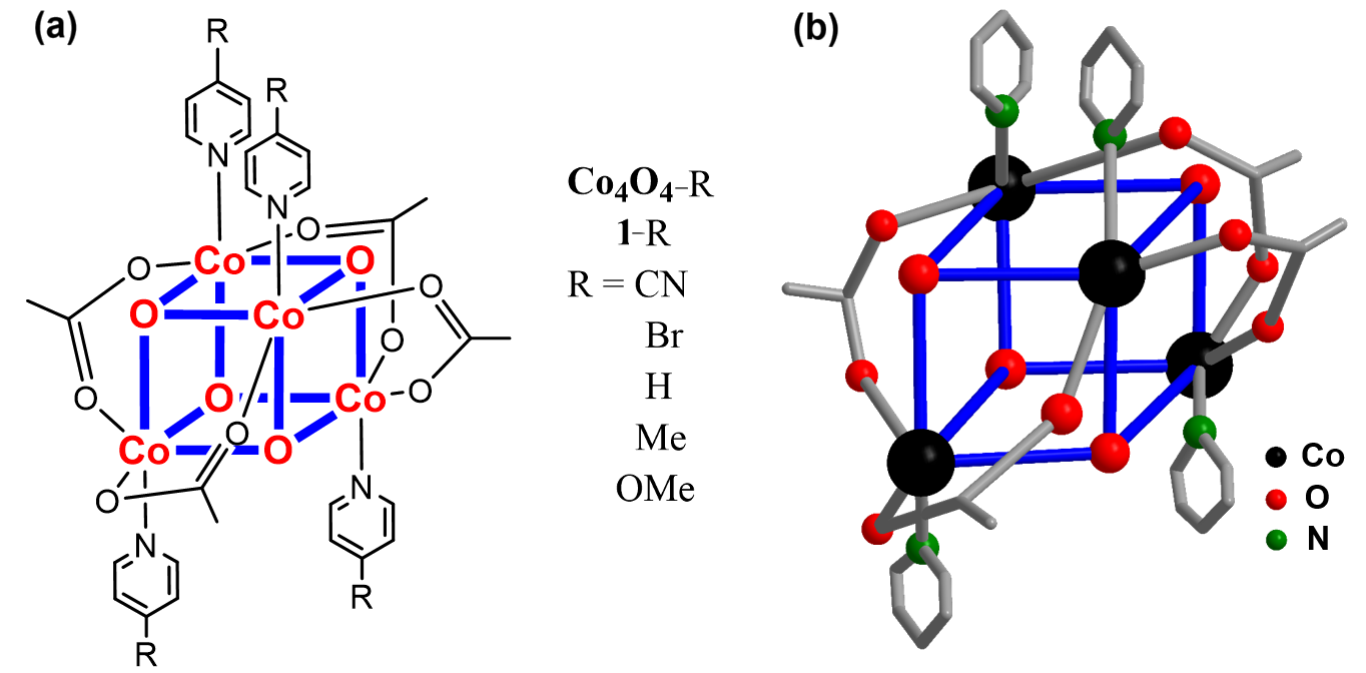
(a) Molecular structures of the Co_4_O_4_ cubane catalysts. (b) Ball-and-stick representation of complex **1**-H; H atoms are omitted for clarity.

Herein, we demonstrate that molecular Co_4_O_4_ cubanes (Figure 1) are readily and precisely manipulated to tune their redox functions through regulating their electronic structures by ligand engineering. The use of electron-withdrawing or donating ligands can easily adjust their catalytic properties for water oxidation and CO_2_ reduction, respectively. For example, organic ligands with strong electron-withdrawing groups (R = CN, Br) enhance their oxidation capability for water oxidation by reducing the overpotential of O–O bond formation. In contrast, the incorporation of electron-donating groups (R = Me, OMe) significantly increases the electronic density at the metal centers, thus affording a Co_4_O_4_ core able to catalyze CO_2_ reduction. This indicates that the change of substituents in the pyridine ligand provides further insight into the factors that affect the redox potential and tailor the catalytic performance. Furthermore, by exploring the structure–function relationship at the molecular level offers a useful guidance for the design and construction of high-performance earth-abundant molecular catalysts.

## Results and Discussion

The molecular Co_4_O_4_ cubanes (**1**-R) were fabricated according to the literature [54], and their identities were confirmed by ^1^H NMR and FTIR spectroscopy (see Supporting Information File 1 for details). Taking catalyst **1**-H as an example, it’s ^1^H NMR spectrum exhibits three sets of peaks at 8.20 (d, 8H), 7.71 (t, 4H) and 7.20 (t, 8H) ppm for the *o*-, *p*-, and *m*-ring protons, respectively, of the equivalent pyridines and the methyl protons of the acetate ligands appear as a sharp singlet at 2.06 (s, 12 H) ppm (see Supporting Information File 1, Figure S1). In the FTIR spectrum, the bands in the region 1530–1538 cm^−1^ are assigned to the ν_asym_(COO) vibration and the stretching vibration of the pyridine ring [54], whereas the band at 1410 cm^−1^ designates to the δ_asym_(CH_3_). The most characteristic feature of the IR data is the appearance of a four-band pattern observed at ≈759, ≈692, ≈634 and ≈574 cm^−1^, corresponding to the “Co_4_O_4_” cubane-like core present in the complex [54]. The XRD patterns for **1**-R cubanes are shown in Supporting Information File 1, Figure S8. Moreover, as shown in Scheme S1 (Supporting Information File 1), all aqueous solutions of **1**-R are transparent, homogeneous and clear, indicative of their similarities in structure. Based on the above analyses and comparison with the data in literatures [54–55], it is concluded that the **1**-R cubanes have been successfully fabricated.

**Figure 2:**
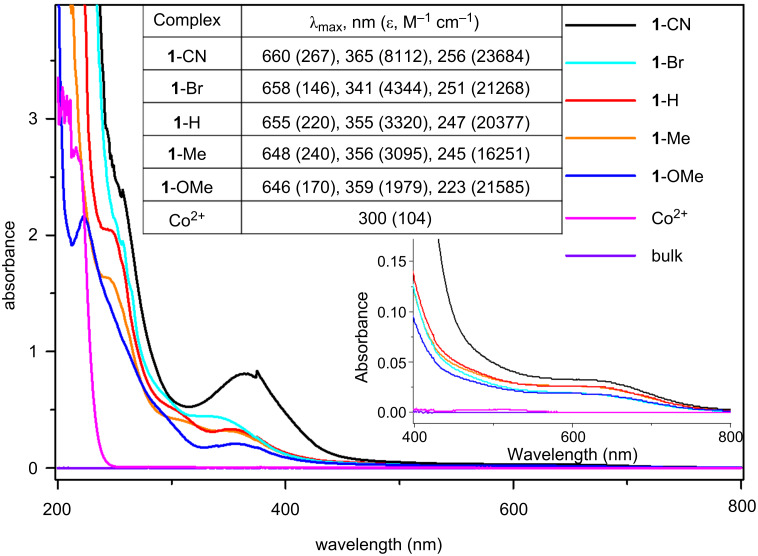
UV–vis absorption spectra of **1**-R in H_2_O based on measurements in 10^−4^ M solution. Inset: scale from 400 nm to 800 nm and the λ_max_ for **1**-R.

Next, to investigate the effect of different ligands on the optical properties of the **1**-R complexes, UV–vis absorption measurements were conducted. As shown in Figure 2, three absorption bands are observed in the UV–vis spectra. The lowest energy absorption appearing as a shoulder at 645 to 660 nm, is associated with the d–d transitions involving ^1^A_1_ → ^1^T_1_ and ^1^A_1_ → ^1^T_2_ for the approximately octahedral Co complex [53–55]. As judged by the observed intensities, the other two bands are attributable to absorptions rather than d–d transitions. The bands in the range of 340 to 365 nm are likely due to a charge-transfer transition involving the μ-O–Co moiety present in these complexes [54–55]. The observed wavelength shift is dependent on the nature of the substituent present in the *p*-position of the pyridine-based ligand. As expected, the incorporation of the electron-withdrawing moiety R = CN reduces the electron densities of the Co centers and thus facilitates the charge-transfer transitions from μ-O atoms to the Co centers, which leads to a modest bathochromic shift from 355 (R = H) to 365 nm, however, with a remarkably enhanced intensity. In addition, the highest energy band between 220–260 nm is believed to be of ligand origin, most probably originating from the π → π* absorption of pyridine [54]. Similarly, a red shift in the order of **1**-CN (256 nm) > **1**-Br (251 nm) > **1**-H (247 nm) > **1**-Me (245 nm) > **1**-OMe (223 nm) is observed based on the increasing electron-withdrawing property of the ligands in these complexes (Figure 2 inset) [55]. This indicates that the different electronic properties have a significant influence on the optical performances, thus underlining the tuning effect of suitably substituted pyridine-based ligands for controlling the catalysts functions.

**Figure 3:**
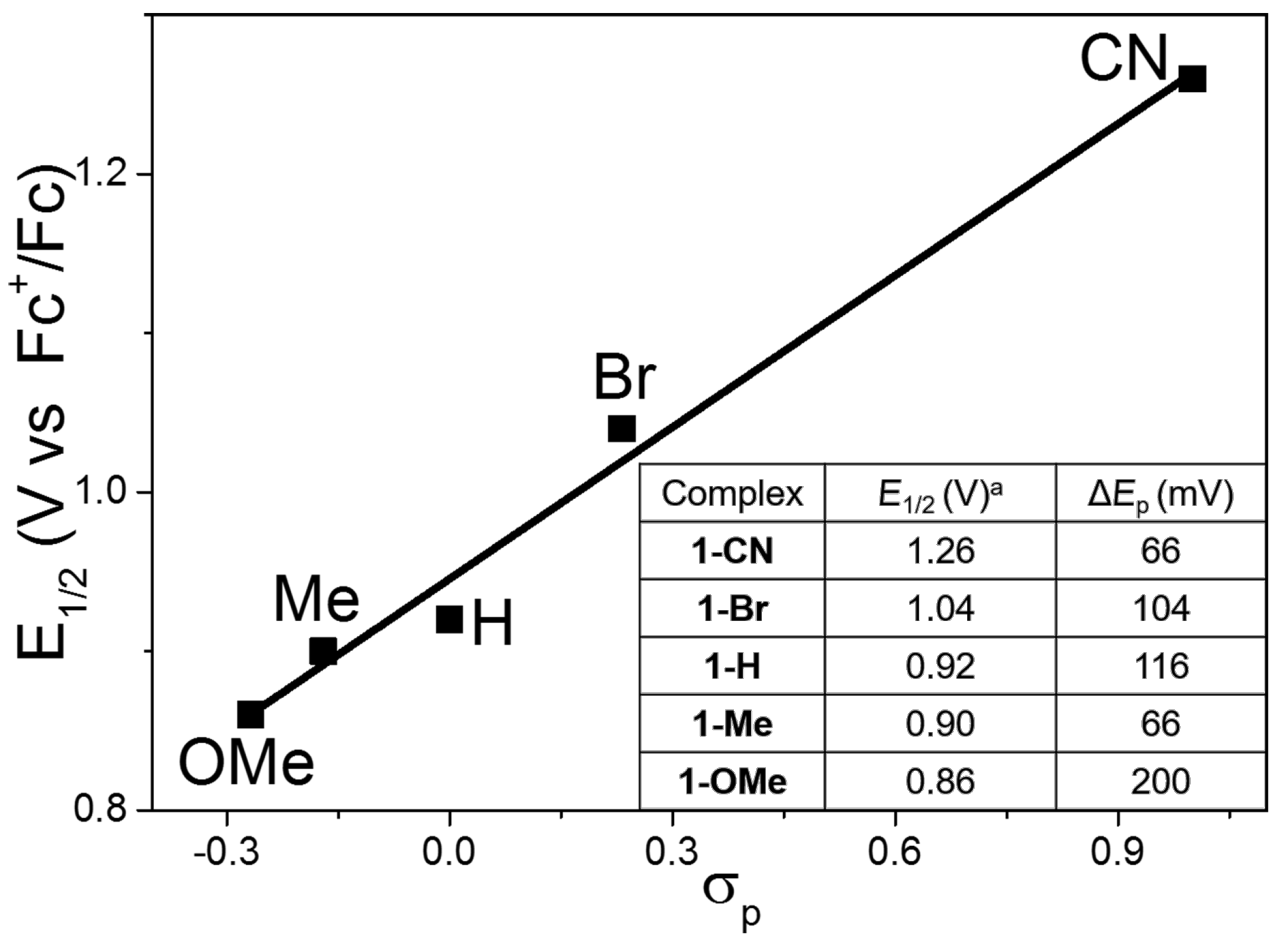
Correlation of Hammett constants σ_p_ for the different ligands with midpoint potentials (*E*_1/2_) in complexes **1**-R. ^a^CV data for **1**-R in MeCN/0.1 M TBAP vs Fc^+^/Fc under saturated Ar atmosphere.

The subsequent cyclic voltammetry (CV) experiments supported the above results, i.e., that the variation of the ligands has a profound effect on the observed redox potentials. Figure 3 displays the plots of the Hammett σ_p_ parameters for the ligands versus the half-wave potentials (*E*_1/2_) for **1**-R complexes, and the potentials increase linearly as a function of σ_p_, giving an indicator of the electronic influence of the substituents on *E*_1/2_. The *E*_1/2_ values for the complexes increase in the following order: **1**-R, R = OMe < Me < H < Br < CN. The Hammett analysis provides a positive slope value, indicating that the *E*_1/2_ value is favored by electron-withdrawing ligands [48,55]. As the ligand becomes more electron withdrawing, the reduced electron density at the metal center makes the Co center in the complex easier to reduce and more difficult to oxidize [55]. Most surprisingly, the potentials could be predicted simply by considering the Hammett σ_p_ values. Therefore, the observed redox potentials reflect a dependence on the electronic properties of the ligand. This again underlines, that the ligands are playing a significant role in the regulation of the redox properties of the **1**-R complexes.

**Figure 4:**
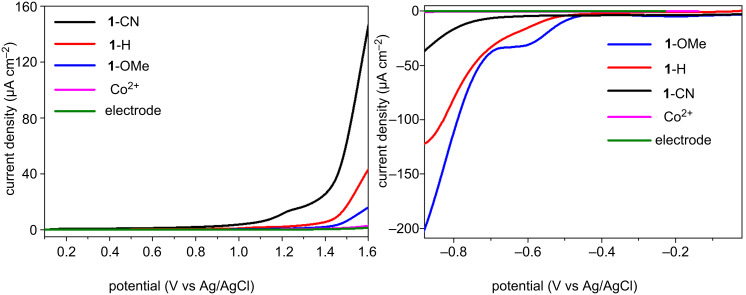
Linear sweep voltammetry of **1**-R (0.3 mM) or Co(NO_3_)_2_·6H_2_O (1.2 mM); (a) at a 100 mV/s scan rate in 0.2 M Na_2_SO_4_, (b) at a 50 mV/s scan rate in MeCN (0.1 M TBAPF_6_) under CO_2_-saturated conditions. The working electrode used was 3 mm diameter glassy carbon electrode, the counter electrode was a platinum foil and the reference electrode was a Ag/AgCl.

To further estimate the impact of ligand substitution, the complexes were analyzed by linear sweep voltammetry (LSV). For this, we chose complexes **1**-CN, **1**-H and **1**-OMe to include ligand substitutions with electron-withdrawing and electron-donating properties (Figure 4). In Figure 4a, an abrupt onset of the catalytic anode current at 0.7 V, 1.0 V and 1.3 V for **1**-CN, **1**-H and **1**-OMe is observed, respectively, which is ascribed to an O_2_ evolution reaction. The ligand substituted with the electron-withdrawing cyano (**CN**) group shows the lowest overpotential for water oxidation activities, exhibiting a much higher current density compared to other cubane complexes and Co^2+^. Meanwhile, we also studied the electrochemical reduction in a CO_2_-saturated system (Figure 4b). It displays that **1**-OMe affords a current density of 200 μA·cm^−2^ at −0.88 V, a 5.5-fold enhancement over **1**-CN (36 μA·cm^−2^). This suggests that the substituted ligand with the electron-donating group is suitable for the electrochemical reduction. It is important to note that ligands with different electronic structures exhibited starkly different activities for redox reaction. The ligand with an electron-withdrawing property favors water oxidation, and the one with electron-donating property is conducive to CO_2_ reduction. Such a favorable electrochemical potential for **1**-R with tunable ligand substitutions suggests their great potential as redox catalysts for water oxidation and CO_2_ reduction reactions.

**Figure 5:**
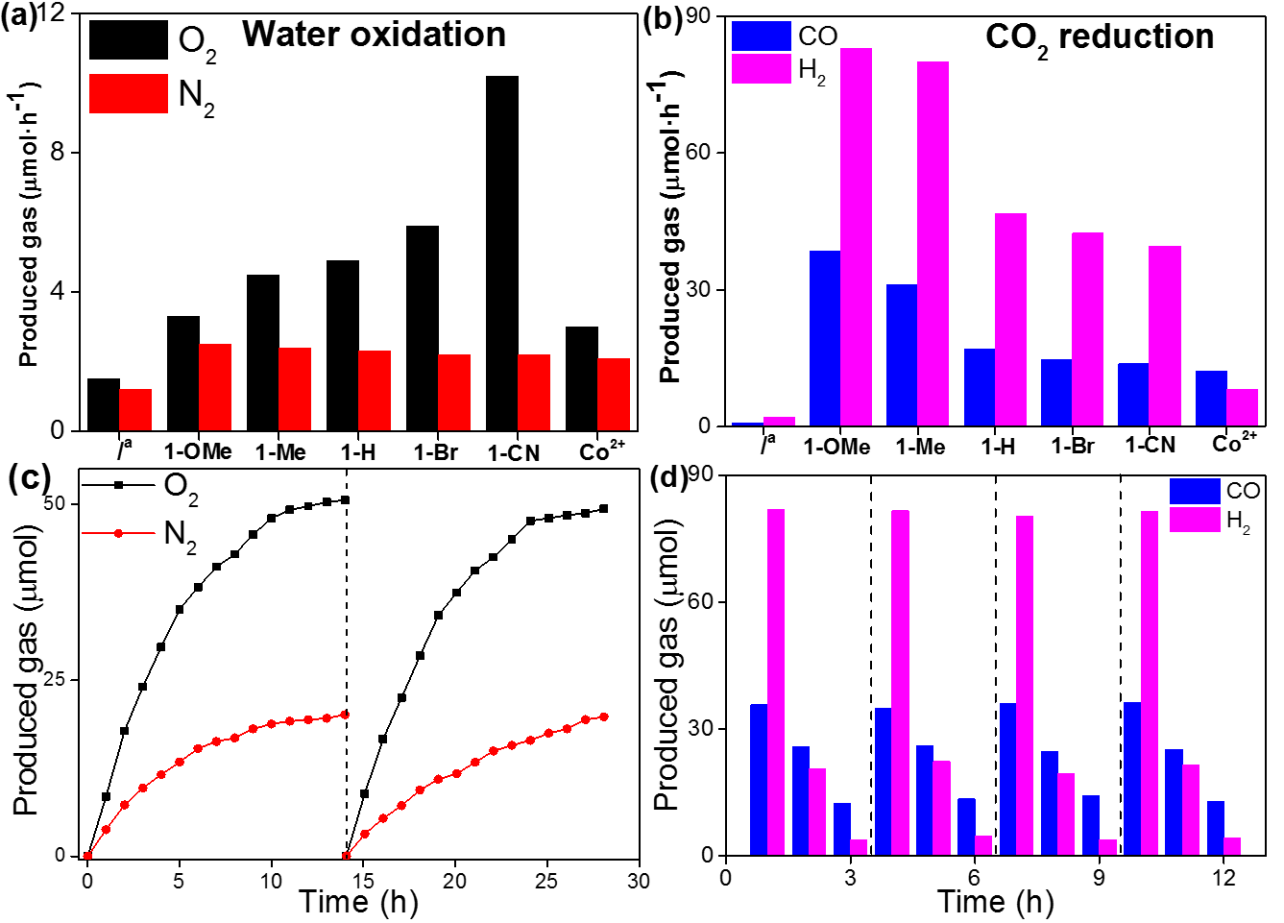
The activity of **1**-R for (a) water oxidation and (b) CO_2_ reduction. (c) Long-time course of water oxidation for **1**-CN under UV–vis light irradiation (λ > 300 nm) in two recycling tests. (d) CO_2_ reduction for **1**-OMe under visible light irradiation (λ > 420 nm) in four recycling tests. ^a^Without **1**-R.

**Figure 6:**
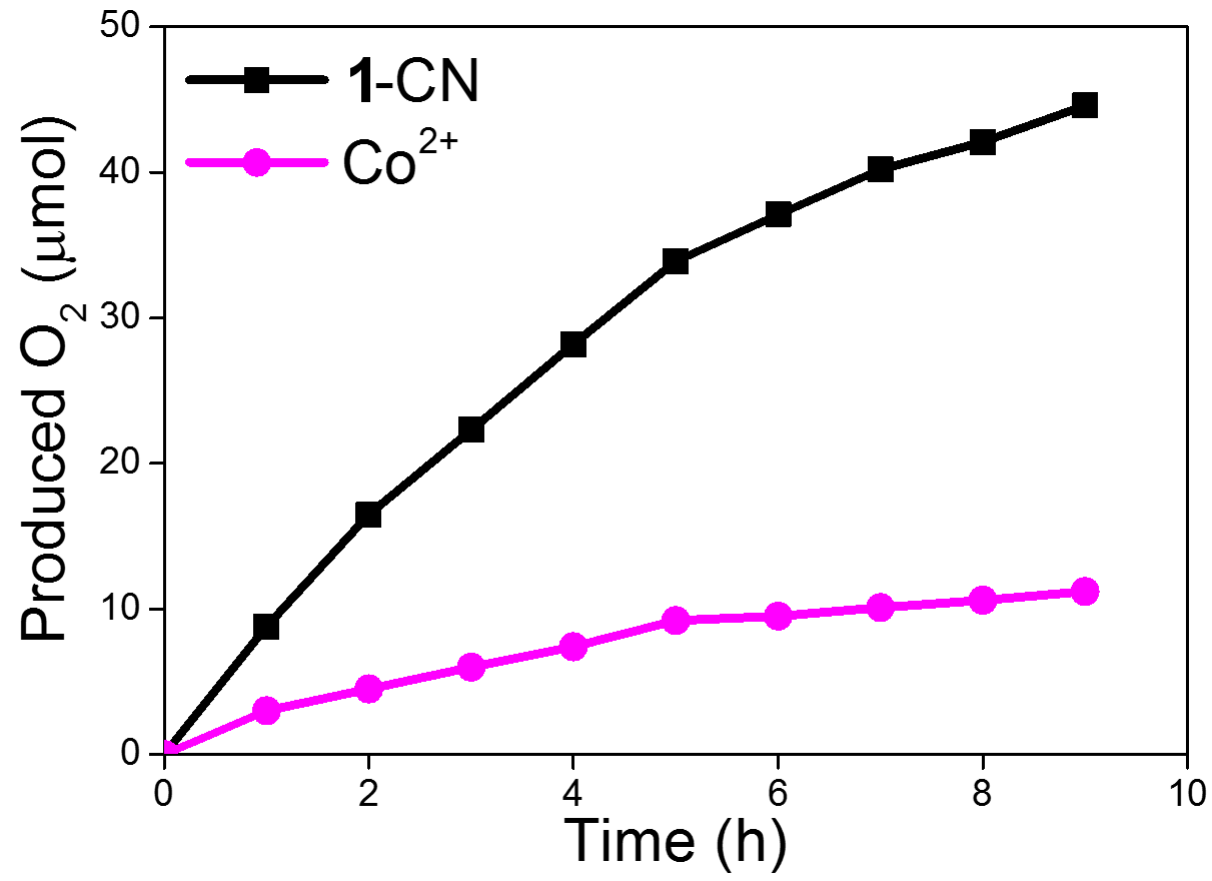
Long-time course of water oxidation for **1**-CN and Co^2+^ under UV–vis light irradiation (λ >300 nm).

Next, we studied the photocatalytic activity of a series of the **1**-R molecular complexes in the water oxidation reaction to release O_2_ gas and CO_2_-to-CO conversion (Figure 5). For the water oxidation, we have chosen carbon nitride [56–60] coupled with the **1**-R molecular complexes to evaluate the oxygen evolution performance. In Figure 5a, without the **1**-R molecular complexes, the O_2_ production rate is rather low (1.2 μmol·h^−1^). However, after introducing the molecular complexes, the oxygen evolution reaction is accelerated, and the reactivity of the reaction is expected to be tuned by the ligand modification, within the order of **1**-CN (10.2 μmol·h^−1^) > **1**-Br (5.9 μmol·h^−1^) > **1**-H (4.9 μmol·h^−1^) > **1**-Me (4.5 μmol·h^−1^) > **1**-OMe (3.3 μmol·h^−1^), which is consistent with the effect of the substituent groups on the pyridine ligand of **1**-R on the electrochemical oxygen evolution. These results indicated that water oxidation is favored by the presence of electron-withdrawing ligands. Additionally, the activity of water oxidation over **1**-R is much higher than that over Co^2+^ ions, which may be due to the effect of the ligand for enhancing the stability of the entire cobalt metal center [48–49]. Furthermore, a long time course of water oxidation for **1**-CN and Co^2+^ are also compared in Figure 6. It is obvious that the overall amount of the produced O_2_ gas for **1**-R is higher than that for Co^2+^. As the reaction time increases, the decreasing trend of O_2_ evolution rate for Co^2+^ is more pronounced than in case of **1**-CN. This can be ascribed to the instability of Co^2+^ and the tendency of this metal to be oxidized to form CoO*_x_* nanoparticles in the reaction mixture (Supporting Information File 1, Figure S7). These results also support the above discussion. The presence of **1**-R with an enhanced electron-withdrawing ability can significantly reduce the overpotential for the O–O bond formation, and thus facilitates water oxidation.

Besides the CO_2_ reduction performance of the molecular complexes was evaluated by cooperation with a ruthenium photosensitizer Ru(bpy)_3_^2+^ (bpy = 2’,2-bipyridine) with visible light irradiation [61–67]. As shown in Figure 5b, the activity of the CO_2_ reaction is reduced with the increase of electron-withdrawing ability of the ligand. In this case, **1**-OMe exhibits the highest CO_2_ photoreduction activity with a CO evolution rate of 38.5 μmol·h^−1^, together with a H_2_ generation rate of 83 μmol·h^−1^. The CO_2_-to-CO conversion rate of **1**-OMe is 2.5-fold enhanced than that of **1**-CN (13.8 μmol·h^−1^). It is found that the introduction of a simple substituent greatly influenced the activity of CO_2_ reduction, that is, the ligand substitution with an electron-donating property is more beneficial for the CO_2_ reduction reaction. The above results demonstrate that the **1**-R molecular complexes are highly active for both water oxidation and CO_2_ reduction reactions, which is in good agreement with the results of the optical, CV and LSV measurements. Importantly, the photoredox functions of the molecular complexes can be modulated deliberately by ligand substitutions with different electronic properties.

The stabilities of the molecular catalysts for photoredox reactions were also examined. Firstly, in 14 h long term water oxidation tests for 2 cycles, the total O_2_ evolution in each run was almost the same (Figure 5c). The gradually reduced reaction rate after about 5 h is ascribed to the deposition of Ag particles on the surface of polymeric carbon nitride (p-C_3_N_4_, PCN), which leads to a light shading effect hindering optical absorption. In the stability test for CO_2_ reduction reactions, no noticeable losses in the yields of CO and H_2_ were observed in 4 cycles (Figure 5d). The deactivation after 3 h reaction in each case is due to photobleaching of the used dye photosensitizer. Moreover, after water oxidation and CO_2_ reduction reactions, the structures of **1**-CN and **1**-OMe were studied by ^1^H NMR spectroscopy, and no obvious changes were observed compared with the fresh samples (Supporting Information File 1, Figure S6).

## Conclusion

In summary, we have developed molecular cubane catalysts with tunable redox potentials through the ligand architectures, which are coupled with the light harvesters (e.g., carbon nitride and Ru(bpy)_3_^2+^) for photocatalytic water oxidation and CO_2_ reduction. The electronic properties of the ligands have a significant effect on the catalysts photoredox reaction. The ligands with electron-withdrawing substituents are beneficial for the water oxidation and the CO_2_ reduction is favored by the presence of electron-donating ligands. The comparative study reported here allows us to scrutinize the interplay between electronic effects and redox potential caused by ligand modifications within the series of Co_4_O_4_ cubane clusters. The ligand modification strategy developed here provides a rational, precise and cost-effective way for the chemical design and synthesis of biomimetic cubane clusters with metal cores (i.e., Co, Mn, and Ni) or even heterobimetallic cores for a wide range of redox applications in catalysis and photosynthesis.

## Experimental

**Materials:** All chemicals are commercially available and were used without further purification. All solutions were prepared with Milli-Q ultrapure water (>18 MΩ) unless otherwise stated.

**Synthesis of p-C_3_N_4_:** p-C_3_N_4_ was synthesized by annealing urea (10 g) at 550 °C for 2 h under the muffle furnace with the ramping rate at 5 °C/min, and the resulted buff powder was collected and denoted as PCN.

**Synthesis of ATCN/p-C_3_N_4_:** The ATCN/p-C_3_N_4_ sample was synthesized according to the literature procedures [68]. 2-Aminothiophene-3-carbonitrile (ATCN, 10 mg) and 10 g urea were mixed with 10 mL pure water, and stirring at room temperature for 12 h and then stirring at 80 °C to remove water. The mixtures were ground into powder and calcined at 550 °C for 2 h under the muffle furnace with the ramping rate at 5 °C/min. The samples thus obtained were denoted as ATCN/PCN.

**Synthesis of Co_4_O_4_(O_2_CMe)_4_(NC_5_H_5_)_4_, 1-H:** Complex **1**-H was synthesized according to the literature procedures [30,48,54]. Typically, to a mixture of Co(NO_3_)_2_·6H_2_O (2.9 g, 10 mmol) and CH_3_CO_2_Na·3H_2_O (2.7 g, 20 mmol) in methanol (30 mL) heated to refluxing temperature, is added pyridine (0.8 mL, 10 mmol) while stirring. Then a portion of 30% hydrogen peroxide (v/v, 5 mL, 50 mmol) is slowly added to the reaction mixture, and stirring under refluxing conditions is continued for 4 h. After cooling to room temperature and reducing the volume, the latter is placed in a separating funnel and CH_2_Cl_2_ added. The pink aqueous phase was discarded, while the dark green organic phase dried over anhydrous Na_2_SO_4_ and filtered. After removal of the solvent, the residue was purified by column chromatography on silica gel with CH_2_Cl_2_/CH_3_OH 15:1 (v/v) as the eluent to afford 1.50 g (70%) of the pure complex as a dark green solid.

**Synthesis of Co_4_O_4_(O_2_CMe)_4_(NC_5_H_4_-OMe)_4_, 1-OMe:** The same procedure as described above was adopted except replacing pyridine with 4-methoxypyridine (1.02 mL, 10 mmol), to afford 2.07 g (85%) of the dark green product.

**Synthesis of Co_4_O_4_(O_2_CMe)_4_(NC_5_H_4_-Me)_4_, 1-Me:** A similar procedure as described above was adopted using 4-methylpyridine (0.98 mL, 10 mmol) to afford 1.82 g (80%) of the dark green product.

**Synthesis of Co_4_O_4_(O_2_CMe)_4_(NC_5_H_4_-Br)_4_, 1-Br:** The same procedure as described above was adopted except replacing pyridine with 4-bromopyridine hydrochloride (1.94 g, 10 mmol) to afford 0.9 g (31%) of the dark green product.

**Synthesis of Co_4_O_4_(O_2_CMe)_4_(NC_5_H_4_-CN)_4_, 1-CN:** The same procedure as described above was adopted except replacing pyridine with 4-cyanopyridine (1.04 mL, 10 mmol) to afford 2.01 g (84%) of the product as dark brown solid.

**Characterization:** The UV–vis absorption spectra were measured on a SHIMADZU UV-1780 spectrometer (Kyoto, Japan). Fourier transform infrared (FTIR) spectra were taken on a thermo Nicolet Nexus 670 FTIR spectrometer with KBr as the diluents. Electrochemical measurements were conducted with a Biologic VSP-300 Electrochemical System in a conventional three electrode cell. The ^1^H NMR experiments were performed on Bruker AVANCE 400M spectrometers. Transmission electron microscopy (TEM) was obtained using a FEI TECNAIG2F20 instrument. Powder X-ray diffraction (XRD) patterns were collected on Bruker D8 Advance diffractometer with Cu K1 radiation (λ = 1.5406 Å).

**Photocatalytic test for water oxidation system** [69]**:** Photocatalytic O_2_ production was carried out in a Pyrex top-irradiation reaction vessel connected to a glass closed gas circulation system. For each reaction, PCN powder (50 mg) was well dispersed in an aqueous solution (100 mL) containing AgNO_3_ (0.17 g) as an electron acceptor, La_2_O_3_ (0.2 g) as a pH buffer agent and **1**-R (0.25 μmol) or Co(NO_3_)_2_·6H_2_O (1.0 μmol). The reaction solution was evacuated several times to remove air completely prior to irradiation with a 300 W xenon lamp with a working current of 15 A (Shenzhen ShengKang Technology Co., Ltd, China, LX300F). The wavelength of the incident light was controlled by applying some appropriate long-pass cut-off filters (λ > 300 nm). The temperature of the reaction solution was maintained at room temperature by a flow of cooling water during the reaction. The evolved gases were analyzed in-situ by gas chromatography equipped with a thermal conductive detector (TCD) and a 5 Å molecular sieves column, using Argon as the carrier gas.

**Photocatalytic test for CO_2_ reduction system** [70]**:** The photocatalytic test was performed in a Schlenk flask (80 mL) under an atmospheric pressure of CO_2_. In the Schlenk flask, the photocatalytic CO_2_ reduction reaction was carried out by dispersing Ru(bpy)_3_^2+^ (7.8 mg) in MeCN (4 mL) containing triethanolamine (TEOA, 1 mL) and **1**-R (0.25 μmol) or Co(NO_3_)_2_·6H_2_O (1.0 μmol). This mixture was subjected to vacuum degassing and then back filling with pure CO_2_ gas. This process was repeated three times, and after the last cycle, the flask was back filled with CO_2_ (1 bar). The temperature of the reaction solution was maintained at 30 °C controlled by a flow of warm water during the reaction. Then, the system was irradiated with a 300 W Xenon lamp with a 420 nm cut-off filter under vigorous stirring. The produced gases (CO and H_2_) were detected using a gas chromatograph equipped with a packed molecular sieves column (TDX-1 mesh 42/10); Argon was used as the carrier gas.

## Supporting Information

File 1 Additional data.

## References

[1] Garrido-Barros P, Gimbert-Suriñach C, Matheu R, Sala X, Llobet A (2017). Chem Soc Rev.

[2] Chu S, Majumdar A (2012). Nature.

[3] Wang S, Guan B Y, Lu Y, Lou X W D (2017). J Am Chem Soc.

[4] Wang S, Wang X (2016). Angew Chem, Int Ed.

[5] Wang S, Guan B Y, Lou X W D (2018). Energy Environ Sci.

[6] Wang S, Guan B Y, Lou X W D (2018). J Am Chem Soc.

[7] Liu Y, Huang B, Xie Z (2018). Appl Surf Sci.

[8] Zhang M, Luo Z, Zhou M, Zhang G, Alamry K A, Taib L A, Asiri A M, Wang X (2017). Appl Catal, B: Environ.

[9] Kärkäs M D, Verho O, Johnston E V, Åkermark B (2014). Chem Rev.

[10] Guo F, Hou Y, Asiri A M, Wang X (2017). Chem Commun.

[11] Zhang G, Lan Z-A, Wang X (2017). Chem Sci.

[12] Chen L, Gu Q, Hou L, Zhang C, Lu Y, Wang X, Long J (2017). Catal Sci Technol.

[13] Pang A, Sun X, Ruan H, Li Y, Dai S, Wei M (2014). Nano Energy.

[14] Ran J, Zhang J, Yu J, Jaroniec M, Qiao S Z (2014). Chem Soc Rev.

[15] Yang J, Wang D, Han H, Li C (2013). Acc Chem Res.

[16] Tachibana Y, Vayssieres L, Durrant J R (2012). Nat Photonics.

[17] Sun J, Zhang J, Zhang M, Antonietti M, Fu X, Wang X (2012). Nat Commun.

[18] Yang P, Wang R, Zhou M, Wang X (2018). Angew Chem, Int Ed.

[19] Yang P, Ou H, Fang Y, Wang X (2017). Angew Chem, Int Ed.

[20] Bonin J, Maurin A, Robert M (2017). Coord Chem Rev.

[21] Wu X, Li F, Zhang B, Sun L (2015). J Photochem Photobiol, C.

[22] Nguyen A I, Wang J, Levine D S, Ziegler M S, Tilley T D (2017). Chem Sci.

[23] Das B, Ezzedinloo L, Bhadbhade M, Bucknall M P, Colbran S B (2017). Chem Commun.

[24] McAlpin J G, Stich T A, Ohlin C A, Surendranath Y, Nocera D G, Casey W H, Britt R D (2011). J Am Chem Soc.

[25] Hodel F H, Luber S (2016). ACS Catal.

[26] Nguyen A I, Ziegler M S, Oña-Burgos P, Sturzbecher-Hohne M, Kim W, Bellone D E, Tilley T D (2015). J Am Chem Soc.

[27] Li X, Siegbahn P E M (2013). J Am Chem Soc.

[28] Song F, Moré R, Schilling M, Smolentsev G, Azzaroli N, Fox T, Luber S, Patzke G R (2017). J Am Chem Soc.

[29] Bi W, Li X, Zhang L, Jin T, Zhang L, Zhang Q, Luo Y, Wu C, Xie Y (2015). Nat Commun.

[30] Wang Y, Li F, Zhou X, Yu F, Du J, Bai L, Sun L (2017). Angew Chem, Int Ed.

[31] Wang Y, Li F, Li H, Bai L, Sun L (2016). Chem Commun.

[32] Schreier M, Luo J, Gao P, Moehl T, Mayer M T, Grätzel M (2016). J Am Chem Soc.

[33] Zhang B, Li F, Yu F, Wang X, Zhou X, Li H, Jiang Y, Sun L (2014). ACS Catal.

[34] Azcarate I, Costentin C, Robert M, Savéant J-M (2016). J Am Chem Soc.

[35] Smith P F, Kaplan C, Sheats J E, Robinson D M, McCool N S, Mezle N, Dismukes G C (2014). Inorg Chem.

[36] Blakemore J D, Crabtree R H, Brudvig G W (2015). Chem Rev.

[37] Kang P, Chen Z, Nayak A, Zhang S, Meyer T J (2014). Energy Environ Sci.

[38] Duan L, Bozoglian F, Mandal S, Stewart B, Privalov T, Llobet A, Sun L (2012). Nat Chem.

[39] Chen Z, Concepcion J J, Brennaman M K, Kang P, Norris M R, Hoertz P G, Meyer T J (2012). Proc Natl Acad Sci U S A.

[40] Huang H, Lin J, Zhu G, Weng Y, Wang X, Fu X, Long J (2016). Angew Chem, Int Ed.

[41] Han X-B, Zhang Z-M, Zhang T, Li Y-G, Lin W, You W, Su Z-M, Wang E-B (2014). J Am Chem Soc.

[42] Zhang G, Zhang M, Ye X, Qiu X, Lin S, Wang X (2014). Adv Mater.

[43] Zhang J, Zhang G, Chen X, Lin S, Möhlmann L, Dołęga G, Lipner G, Antonietti M, Blechert S, Wang X (2012). Angew Chem, Int Ed.

[44] Artero V, Chavarot-Kerlidou M, Fontecave M (2011). Angew Chem, Int Ed.

[45] Sartorel A, Bonchio M, Campagna S, Scandola F (2013). Chem Soc Rev.

[46] La Ganga G, Puntoriero F, Campagna S, Bazzan I, Berardi S, Bonchio M, Sartorel A, Natali M, Scandola F (2012). Faraday Discuss.

[47] Polarz S, Orlov A V, van den Berg M W E, Driess M (2005). Angew Chem, Int Ed.

[48] Berardi S, La Ganga G, Natali M, Bazzan I, Puntoriero F, Sartorel A, Scandola F, Campagna S, Bonchio M (2012). J Am Chem Soc.

[49] Evangelisti F, Güttinger R, Moré R, Luber S, Patzke G R (2013). J Am Chem Soc.

[50] Yin Q, Tan J M, Besson C, Geletii Y V, Musaev D G, Kuznetsov A E, Luo Z, Hardcastle K I, Hill C L (2010). Science.

[51] Wasylenko D J, Ganesamoorthy C, Borau-Garcia J, Berlinguette C P (2011). Chem Commun.

[52] Dogutan D K, McGuire R, Nocera D G (2011). J Am Chem Soc.

[53] Das B K, Chakrabarty R (2011). J Chem Sci.

[54] Chakrabarty R, Bora S J, Das B K (2007). Inorg Chem.

[55] Chakrabarty R, Sarmah P, Saha B, Chakravorty S, Das B K (2009). Inorg Chem.

[56] Wang X, Maeda K, Thomas A, Takanabe K, Xin G, Carlsson J M, Domen K, Antonietti M (2009). Nat Mater.

[57] Wang X, Chen X, Thomas A, Fu X, Antonietti M (2009). Adv Mater.

[58] Lin Z, Wang X (2013). Angew Chem, Int Ed.

[59] Zhang J, Zhang M, Sun R-Q, Wang X (2012). Angew Chem, Int Ed.

[60] Cui Y, Ding Z, Fu X, Wang X (2012). Angew Chem, Int Ed.

[61] Lin J, Ding Z, Hou Y, Wang X (2013). Sci Rep.

[62] Wang S, Yao W, Lin J, Ding Z, Wang X (2014). Angew Chem, Int Ed.

[63] Wang S, Ding Z, Wang X (2015). Chem Commun.

[64] Kuriki R, Matsunaga H, Nakashima T, Wada K, Yamakata A, Ishitani O, Maeda K (2016). J Am Chem Soc.

[65] Kuriki R, Yamamoto M, Higuchi K, Yamamoto Y, Akatsuka M, Lu D, Yagi S, Yoshida T, Ishitani O, Maeda K (2017). Angew Chem, Int Ed.

[66] Kuriki R, Sekizawa K, Ishitani O, Maeda K (2015). Angew Chem, Int Ed.

[67] Wang S, Hou Y, Wang X (2015). ACS Appl Mater Interfaces.

[68] Zhang M, Wang X (2014). Energy Environ Sci.

[69] Zhang G, Zang S, Lin L, Lan Z-A, Li G, Wang X (2016). ACS Appl Mater Interfaces.

[70] Lin J, Pan Z, Wang X (2014). ACS Sustainable Chem Eng.

