# Supplementary material for: Bioinspired cobalt cubanes with tunable redox potentials for photocatalytic water oxidation and CO2 reduction
Source: Beilstein J Org Chem. 2018 Sep 5;14:2331–9. doi: 10.3762/bjoc.14.208 (PMC6142774; doi:10.3762/bjoc.14.208)
Supplement: File 1 — Additional data. [file Beilstein_J_Org_Chem-14-2331-s001.pdf]

Supporting Information

for

Bioinspired cobalt cubanes with tunable redox potentials for photocatalytic water oxidation and CO_2 reduction

Zhishan Luo, Yidong Hou, Jinshui Zhang, Sibo Wang, and Xinchen Wang*

Address: State Key Laboratory of Photocatalysis on Energy and Environment,

College of Chemistry, Fuzhou University, Fuzhou 350002, China.

Email: Xinchen Wang - xcwang@fzu.edu.cn

* Corresponding author

Additional data

1. Supplementary methods

Electrochemical measurements for linear sweep voltammograms: 1-R (0.3 mM) or $Co(NO_3)_2 \cdot 6H_2O$ (1.2 mM) at a 100 mV/s scan rate in 0.2 M Na_2SO_4 for the oxidation part and in the reduction part, the scan rate is 50 mV/s in MeCN (0.1 M $TBAPF_6$) under Ar or CO_2-saturated atmosphere. The working electrode used was a 3 mm diameter glassy carbon electrode, the counter electrode was a platinum foil and as the reference electrode Ag/AgCl was used.

Electrochemical measurements for cyclic voltammetry: 1-R (0.3 mM) in MeCN/0.1 M TBAP vs. Fc^+/Fc at a 50 mV/s under Ar-saturated atmosphere. As the working and counter electrodes glassy carbon (3 mm diameter) was used, the reference electrode was Ag wire.

The reaction conditions of stability experiments for water oxidation: Photocatalytic O_2 production was carried out in a Pyrex top-irradiation reaction vessel connected to a glass closed gas circulation system. For a typical reaction, 50 mg PCN powder was well dispersed in an aqueous solution (100 mL) containing $AgNO_3$ (0.17 g), La_2O_3 (0.2 g) and **1**-CN (0.25 μmol) or $Co(NO_3)_2 \cdot 6H_2O$ (1.0 μmol) under UV–vis light irradiation (λ > 300 nm). After 14 hours, the solution was filtered by centrifugation, the precipitates washed with 20 mL pure water and the solution collected. Compound **1**-CN was obtained by rotary evaporation and purified by column chromatography for the next text cycle. Fresh PCN, $AgNO_3$ and La_2O_3 were added to the obtained **1**-CN for the next long time course of water oxidation.

The reaction conditions of stability experiment for CO_2 reduction: The photocatalytic test was performed in a Schlenk flask (80 mL) under an atmospheric pressure of CO_2. In the Schlenk flask, the photocatalytic CO_2 reduction reaction was carried out by dispersing $[Ru(bpy)_3]Cl_2 \cdot 6H_2O$ (7.8 mg) in MeCN (4 mL) containing triethanolamine (TEOA, 1 mL) and **1**-R (0.25 μmol) or

$Co(NO_3)_2·6H_2O$ (1.0 μmol). The mixture was subjected to vacuum degassing and back filling with pure CO_2 gas. This process was repeated three times, and after the last cycle, the flask was back-filled with CO_2 (1 bar). The temperature of the reaction solution was maintained at 30 °C controlled by a flow of warm water during the reaction. Then, the system was irradiated with a 300 W Xenon lamp with a 420 nm cut-off filter under vigorous stirring. Every 1 hour, the flask was evacuated and refilled with CO_2 (1 bar); every 3 hours $[Ru(bpy)_3]Cl_2·6H_2O$ (6.0 mg), MeCN (3 mL) and TEOA (0.8 mL) were added.

The purification process after water oxidation for 1-CN: In a typical case, after the photocatalytic test with PCN, **1**-CN was obtained by rotary evaporation. After the removal of the solvent, the residue was purified by column chromatography using CH_2Cl_2/CH_3OH 15:1 (v/v) as the eluent to afford pure **1**-CN.

The purification process after CO_2 reduction for 1-OMe: The reaction was done with the ATCN/PCN instead of $[Ru(bpy)_3]Cl_2·6H_2O$ system. In a typical case, after the photocatalytic test with ATCN/PCN, **1**-OMe was obtained rotary evaporation. After the removal of the solvent, the residue was purified by column chromatography using CH_2Cl_2/CH_3OH 15:1 (v/v) as the eluent to afford pure **1**-OMe.

2. ^{1}H NMR and FTIR data

$Co_4O_4(O_2CMe)_4(NC_5H_5)_4$, 1-H: ^{1}H NMR (400 MHz, D_2O): 8.20 (d,8H), 7.71 (t,4H), 7.20 (t,8H), 2.06 (s, 12H). FT-IR (KBr, pollet; wavenumber, cm^{-1}): 3417 (w, br), 3110 (w), 3077 (w), 2927 (w), 2850 (w) 1608 (m), 1535 (m), 1483 (s), 1452 (s), 1415 (vs), 1342 (w), 1213 (m), 1157 (m), 1074 (m), 1043 (w), 1016 (w), 759 (s), 692 (s), 634 (s), 574 (s) and 453 (w).

$Co_4O_4(O_2CMe)_4(NC_5H_4\text{-}OMe)_4$, 1-OMe: ^1H NMR (400 MHz, CD_3CN): 8.12 (d, 8H), 6.69 (d,8H), 3.85 (s, 12H), 1.98 (s, 12H). FT-IR (KBr, pollet; wavenumber, cm^{-1}): 3019 (w), 2843 (w), 1612 (s), 1565 (m), 1542 (m, br), 1509 (s), 1410 (s, br), 1380 (s), 1349 (m), 1294 (s), 1203 (s), 1065 (m), 1034 (s), 1013 (m), 822 (m), 700 (m), 668 (m), 633 (m).

$Co_4O_4(O_2CMe)_4(NC_5H_4\text{-}Me)_4$, 1-Me: ^1H NMR (400 MHz, CD_3CN): 8.17 (d, 8H), 6.96 (d,8H), 3.85 (s, 12H), 2.35 (s, 12H), 1.96 (s, 12H). FT-IR (KBr, pollet; wavenumber, cm^{-1}): 3079 (w), 2953 (w), 2923 (w),1712 (w), 1665 (m), 1540 (s, br), 1503 (s), 1410 (s, br), 1380 (s), 1339 (m), 1244 (w), 1223 (w), 1205 (m), 1064 (w), 1033 (w), 812 (m), 720 (w), 688 (m), 633 (m).

$Co_4O_4(O_2CMe)_4(NC_5H_4\text{-}Br)_4$, 1-Br: ^1H NMR (400 MHz, CD_3CN): 8.19 (d, 8H), 7.40 (d,8H), 1.98 (s, 12H). FT-IR (KBr, pollet; wavenumber, cm^{-1}): 3099 (w), 3029 (w), 2925 (w),1716 (w), 1635 (m), 1590 (s), 1533 (s, br), 1480 (s), 1355 (s, br), 1339 (m), 1204 (m), 1093 (w), 1055 (w), 814 (m), 701 (m), 638 (m).

$Co_4O_4(O_2CMe)_4(NC_5H_4\text{-}CN)_4$, 1-CN: ^1H NMR (400 MHz, CD_3CN): 8.62 (d, 8H), 7.50 (d, 8H), 2.01 (s, 12H). FT-IR (KBr, pollet; wavenumber, cm^{-1}): 3113 (w), 3058 (w), 2926 (w), 2237 (w), 1612 (w), 1533 (s), 1493 (m), 1417 (s), 1385 (s), 1343 (w), 1211 (w), 1065 (w), 1031 (w), 834 (m), 793 (w), 702 (m), 635 (m), 587 (m), 572 (m), 562 (m).

3. Supplementary Scheme

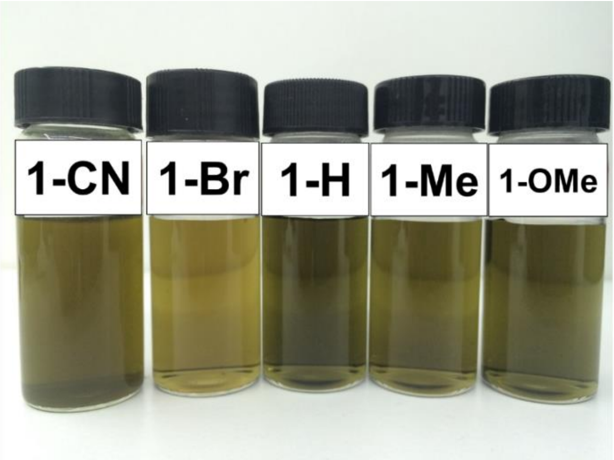

Scheme S1: Solution of **1**-R complexes in water.

4. Supplementary Figures

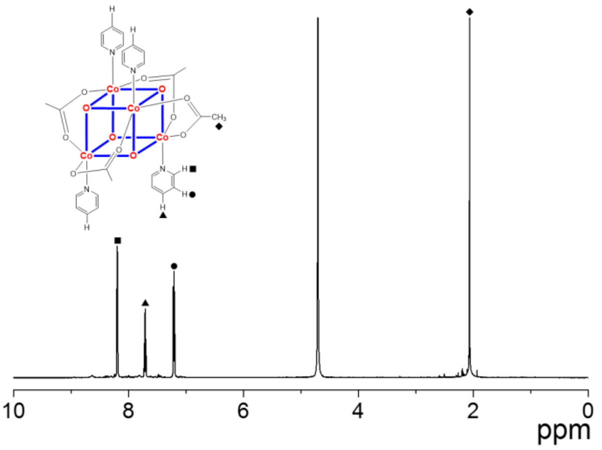

Figure S1: ^{1}H NMR spectrum of **1**-H in D_2O (inset: the structure of **1**-H).

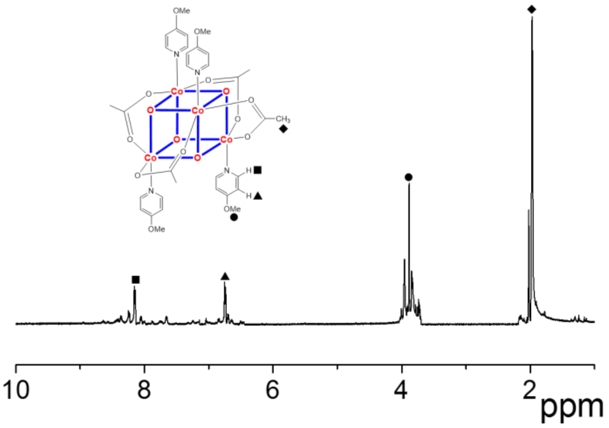

Figure S2: ^{1}H NMR spectrum of **1**-OMe in CD_3CN (inset: the structure of **1**-OMe).

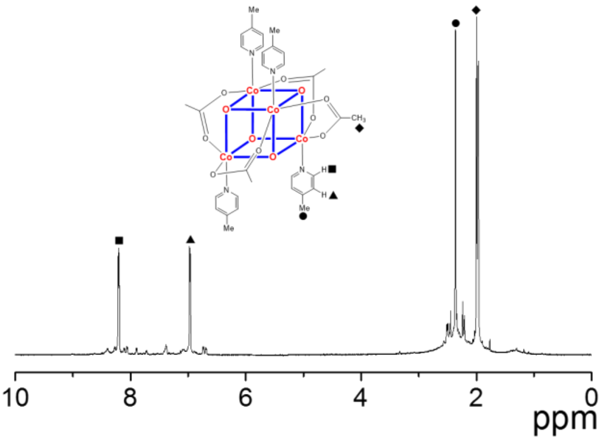

Figure S3: ^{1}H NMR spectrum of **1**-Me in CD_3CN (inset: the structure of **1**-Me).

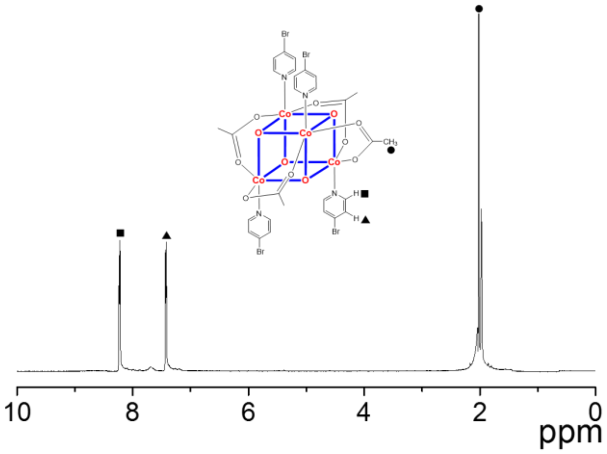

Figure S4: ^{1}H NMR spectrum of **1**-Br in CD_3CN (inset: the structure of **1**-Br).

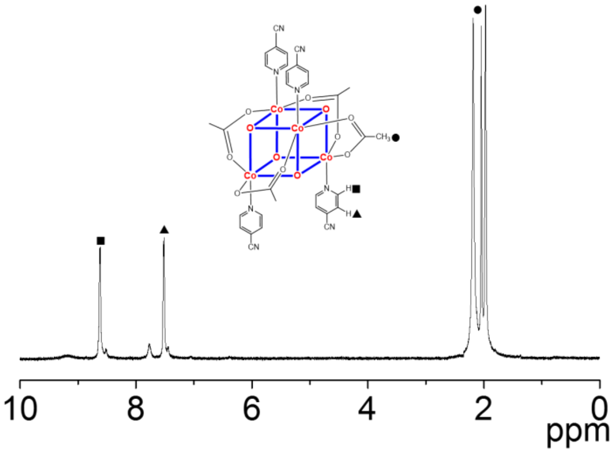

Figure S5: ^{1}H NMR spectrum of **1**-CN in CD_3CN (inset: the structure of **1**-CN).

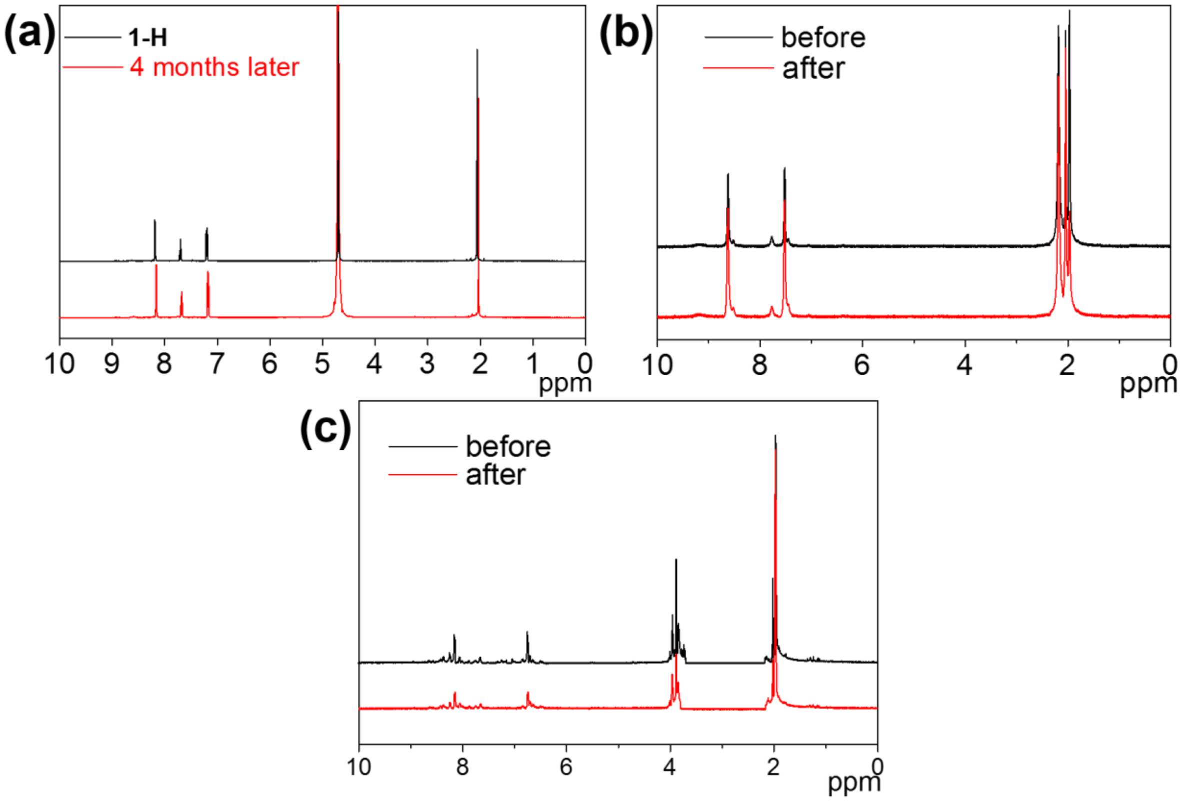

Figure S6: ^{1}H NMR spectrum of (a) **1**-H before and after 4 months storage; (b) **1**-CN before and after water oxidation; (c) **1**-OMe before and after CO_2 reduction.

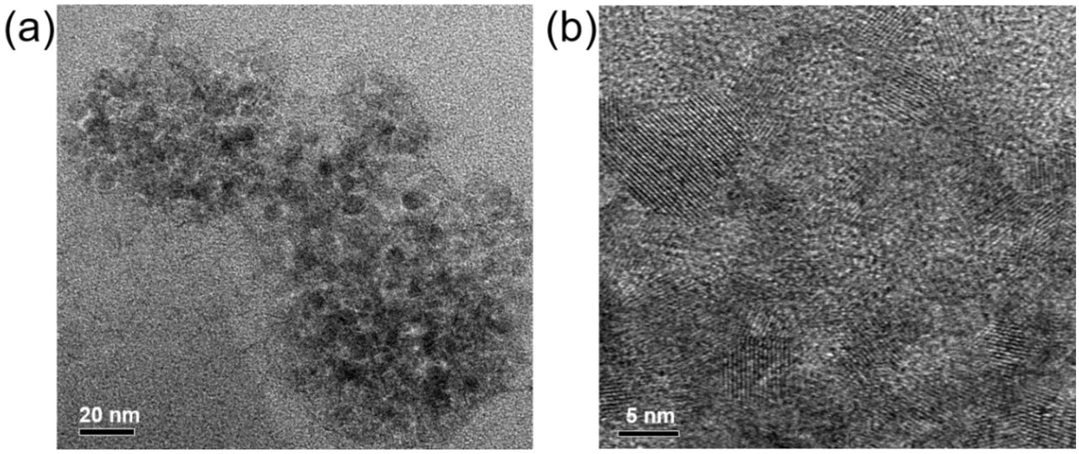

Figure S7: (a) TEM and (b) HRTEM of of Co^{2+} after photocatalytic water oxidation.

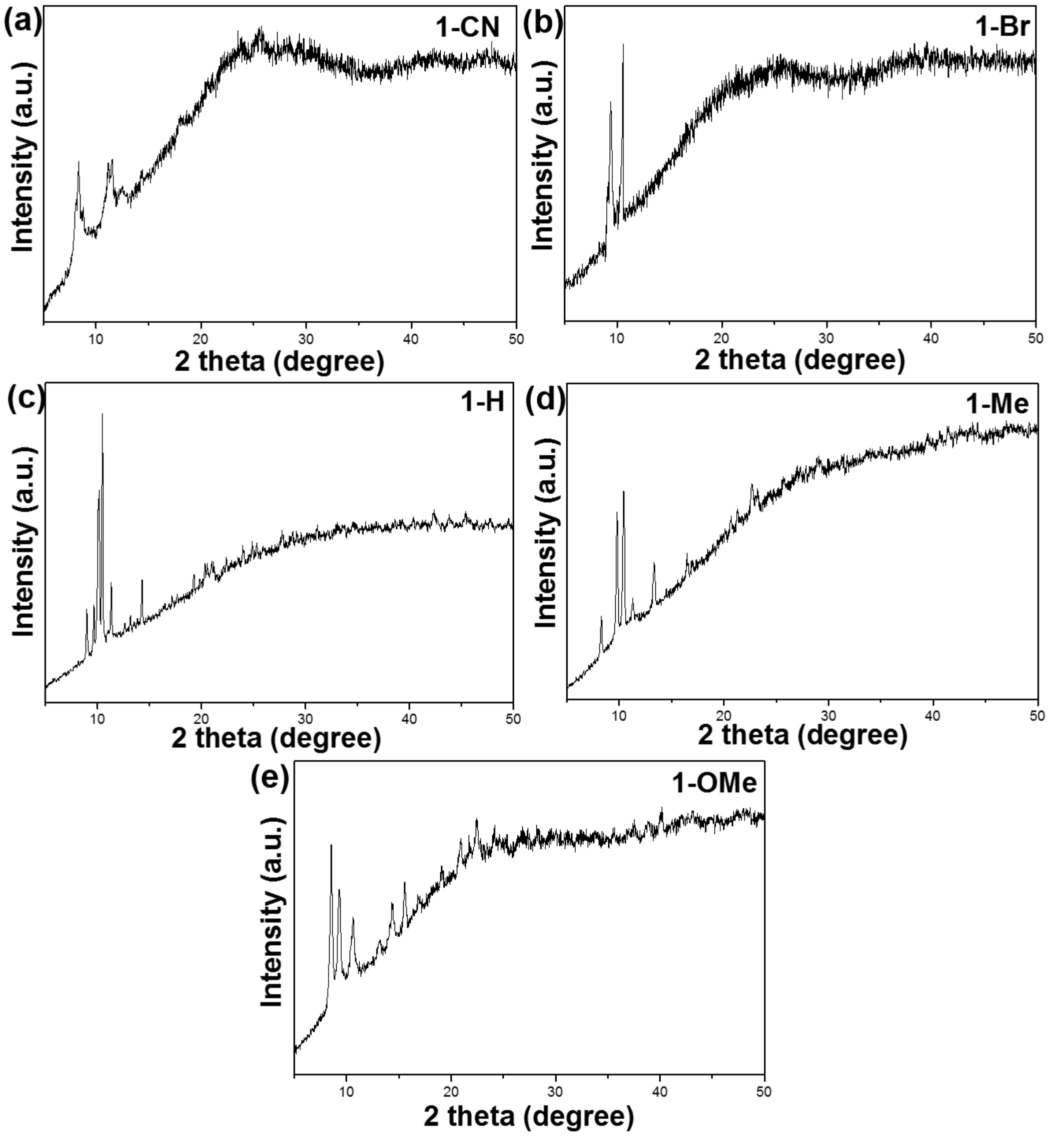

Figure S8: The XRD patterns for (a) **1**-CN, (b) **1**-Br, (c) **1**-H, (d) **1**-Me, and (e) **1**-OMe.
